# Coeur en sabot

**DOI:** 10.5830/cvja-2010-026

**Published:** 2010-08

**Authors:** FAHAD AZIZ, MARY ABED

**Affiliations:** Department of Internal Medicine, MSS M, Jersey City Campus, New Jersey, USA; Department of Internal Medicine, MSS M, Jersey City Campus, New Jersey, USA

**Keywords:** uncorrected TO, congestive heart failure, longevity

## Abstract

In tetralogy of Fallot (TOF), the most common form of cyanotic congenital heart disease, only a few patients reach adulthood without surgical correction. We present a case of a woman with TOF who was diagnosed at the age of 39 when she presented with features of congestive heart failure. The main factor contributing to her longevity included the slow development of her pulmonary artery stenosis together with left ventricular hypertrophy. Less than 3% of all patients with uncorrected TOF survive beyond their 40s but late operative repair is still a valuable option. This case provides an insight into the late outcome of an older patient with uncorrected TOF.

## Introduction

More than 40 years have passed since the first successful repair of tetralogy of Fallot (TOF), and currently, excellent results for the repair of most TOF variants have been achieved. Although most patients undergo radical repair during infancy and childhood, patients remaining undiagnosed and untreated until adulthood can still be treated. These patients have either a previous palliative or natural collateral circulation to the lung or a mild form of right ventricular outflow tract (RVOT) obstruction. A few case reports of patients with TOF surviving until their eighth decade of life have been reported.^1^

The survival data of patients with TOF who have died without surgical treatment reveal that 66% lived to the first year of life, 56% to two years, 49% to three years, and 25% to 10 years of age. Thereafter, the hazard function (or the instantaneous risk of death at any given age) remains essentially constant at 6.4% per year, so that only 3% of persons born with TOF are alive at 40 years of age. The natural history of the disease is influenced by the severity of the anatomical defect, primarily the severity of the pulmonary stenosis.^2^

## Case report

A 39-year-old female patient was admitted to our hospital with dyspnoea as the main symptom. She had had a murmur since childhood but it was never investigated, and she was not able to play as a child due to dyspnoea. Her dyspnoea had increased in the previous two weeks, as had her generalised body oedema.

**Fig. 1.**
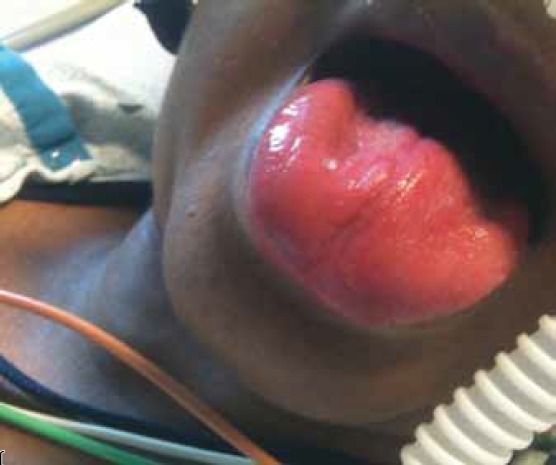
Central cyanosis.

On physical examination, she was found to have blood pressure of 90/60 mmHg with a regular heart rate of 66 beats per min. Her jugular vein distention was raised about 15 cm above the sternal angle with a prominent A wave. She had central and peripheral cyanosis (Fig. 1). The pericardial examination was significant for the visible apex beat pulsations in the fifth intercostal space in the mid-axillary line and there were also visible pulsations along the parasternal border and second intercostal space. The apex beat was palpable in the fifth intercostal space with ill-sustained heave. The second heart sound was palpable in the second intercostal space.

**Fig. 2.**
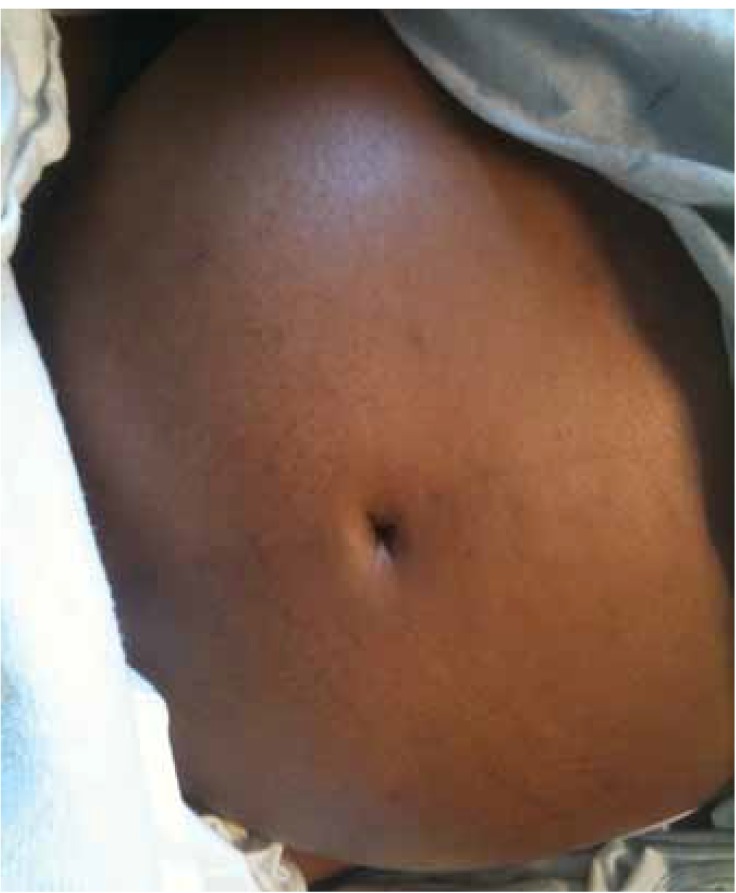
Anterior abdominal wall oedema.

**Fig. 3.**
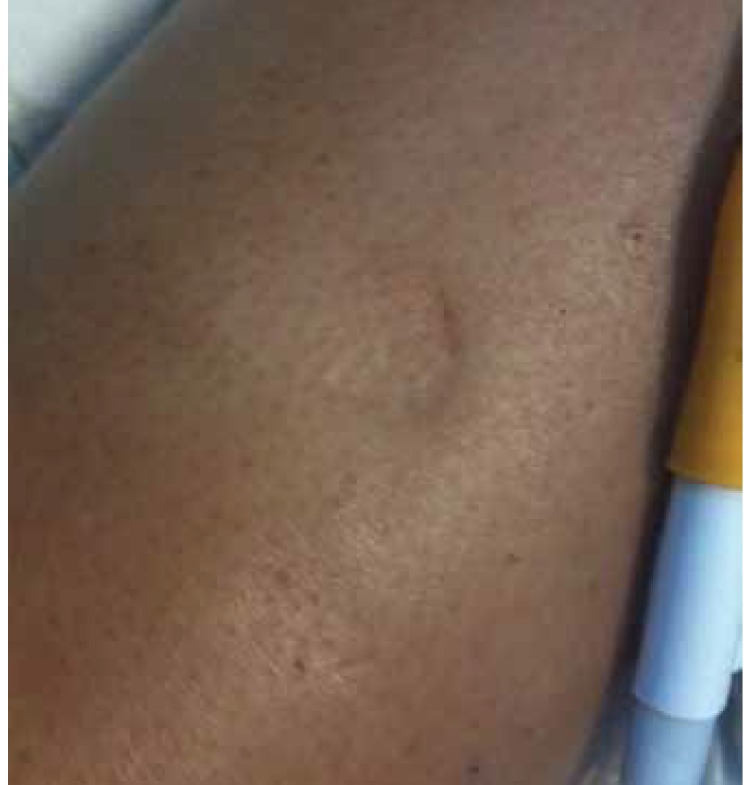
Oedema extending up to the upper thigh.

Auscaltation of the heart revealed S1, S2 and S4 along with a systolic murmur of grade 4/6 with maximum intensity in the left second intercostal space. Lung auscultation revealed bilateral crackles up to one-third of the chest bilaterally. The abdominal examination was significant for tender hepatomegaly, which was four fingers below the costal margins, and the total hepatic span was measured to be 19 cm. She had bilateral pedal oedema extending up to the lower abdomen and involving the external genitalia (Figs 2, 3). She also had grade II clubbing bilaterally.

**Fig. 4.**
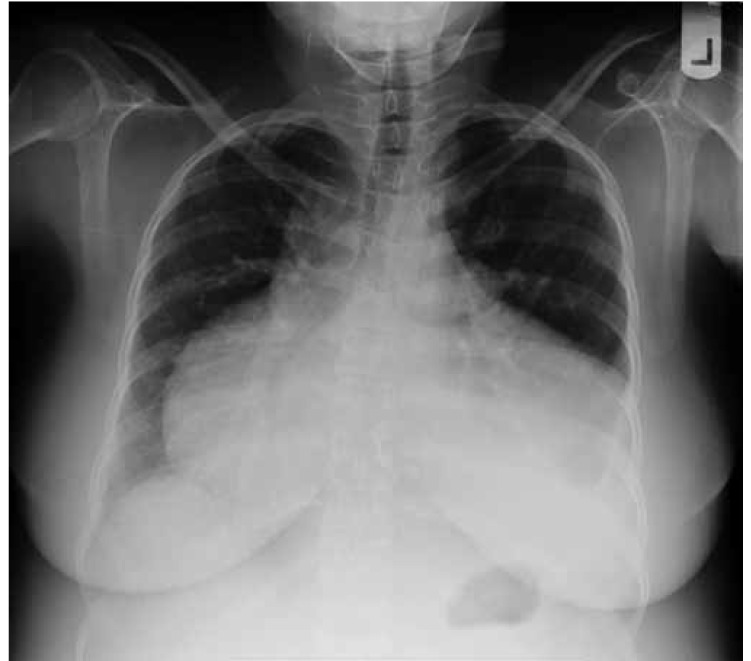
Chest X-ray postero-anterior view.

**Fig. 5.**
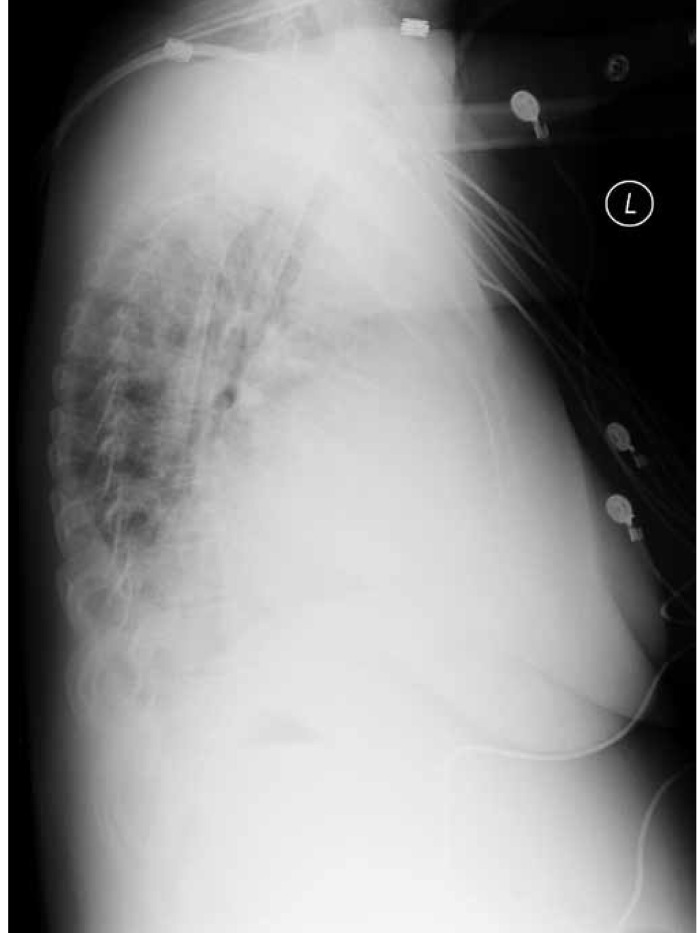
Chest X-ray lateral view.

**Fig. 6.**
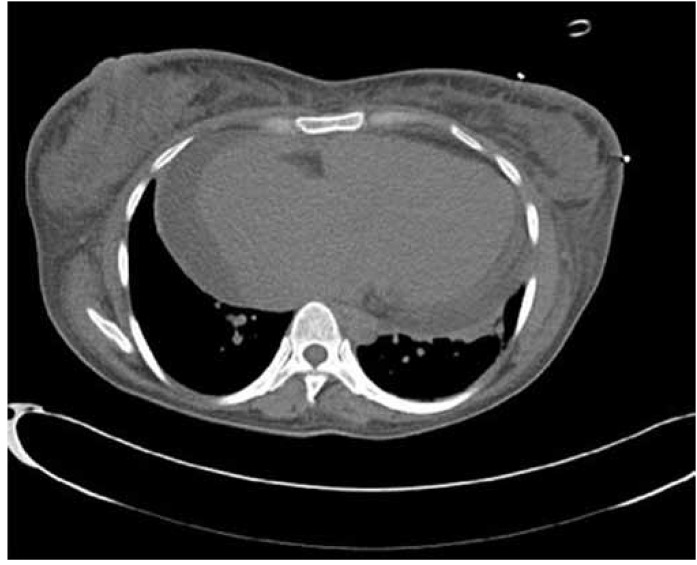
Non-enhanced CT scan of the chest.

The chest X-ray showed a large boot-shaped heart (Figs 4–6). The patient was admitted to the critical care unit with a diagnosis of congestive heart failure and she was started on empirical therapy. The next day an echocardiogram was done, which showed marked left ventricular hypertrophy, a dilated left and right atrium and severe tricuspird regurgitation associated with severe pulmonary artery stenosis, ventricular septal defect (VSD) and moderate to severe right ventricular hypertrophy. On the basis of these findings, a diagnosis of tetrology of Fallot was made and the patient was referred for corrective surgery.

## Discussion

The unusual longevity in some cases of tetralogy of Fallot is probably related to comparatively favorable anatomical abnormalities. Higgins,^3^ after reviewing the literature, concluded that longevity is determined predominantly by the early development of collateral circulation to the lungs and the progressive narrowing with age of the initially mild infundibular stenosis.

In adults with TOF there are several differences noted from the classic description of this lesion in children. These include a very large VSD with greater frequency of combined infundibular and valvular pulmonary stenosis. Usually adults with TOF present with congestive heart failure in 33% of cyanotic and 38% of acyanotic patients; characteristic chest pain occurring in early adult life; a symptomatic quiescent period during the second decade of life; normal or increased pulmonary vasculature in 42% of cyanotic patients and absence of this hypervascularity in 70% of acyanotic patients; and ECG evidence of right bundle branch block in 58% and tall peaked P waves in 66% of cyanotic patients, and predominantly right ventricular hypertrophy in 46% of acyanotic patients.^3^

Special considerations for anaesthetic and post-operative management in adults with TOF are: fibrosed and stiff right ventricle, a tendency to develop congestive heart failure and bilateral pleural effusions, and the presence of major aortopulmonary collateral arteries (MAPCAs). The progressive narrowing of the initially mild pulmonary artery stenosis in our patient undoubtedly contributed to her longevity and then deterioration of her symptoms later in her life.

Post-operatively, these patients are at risk of developing pulmonary oedema, because of increased pulmonary vascularity and large MAPCAs and may require prolonged ventilation with positive end-expiratory pressure (PEEP). Congestive cardiac failure leads to the development of bilateral pleural effusions that may appear multiple times and may take weeks to months to resolve completely.

Although complete repair of TOF in the neonate is associated with excellent intermediate-term survival,^4^ the overall survival of surgically treated adult patients with TOF is also acceptable. The major benefit of complete repair in adults is their functional improvement. On the other hand, late complications closely related to chronic hypoxia, such as arrhythmia and ventricular dysfunction, might require more careful follow up after the surgical correction.^5^

There are a limited number of cases of patients who have lived with untreated TOF until adulthood. This case serves as a reminder of TOF as a differential diagnosis in cynotic patients with congestive heart failure, and its unique manifestations and management.
